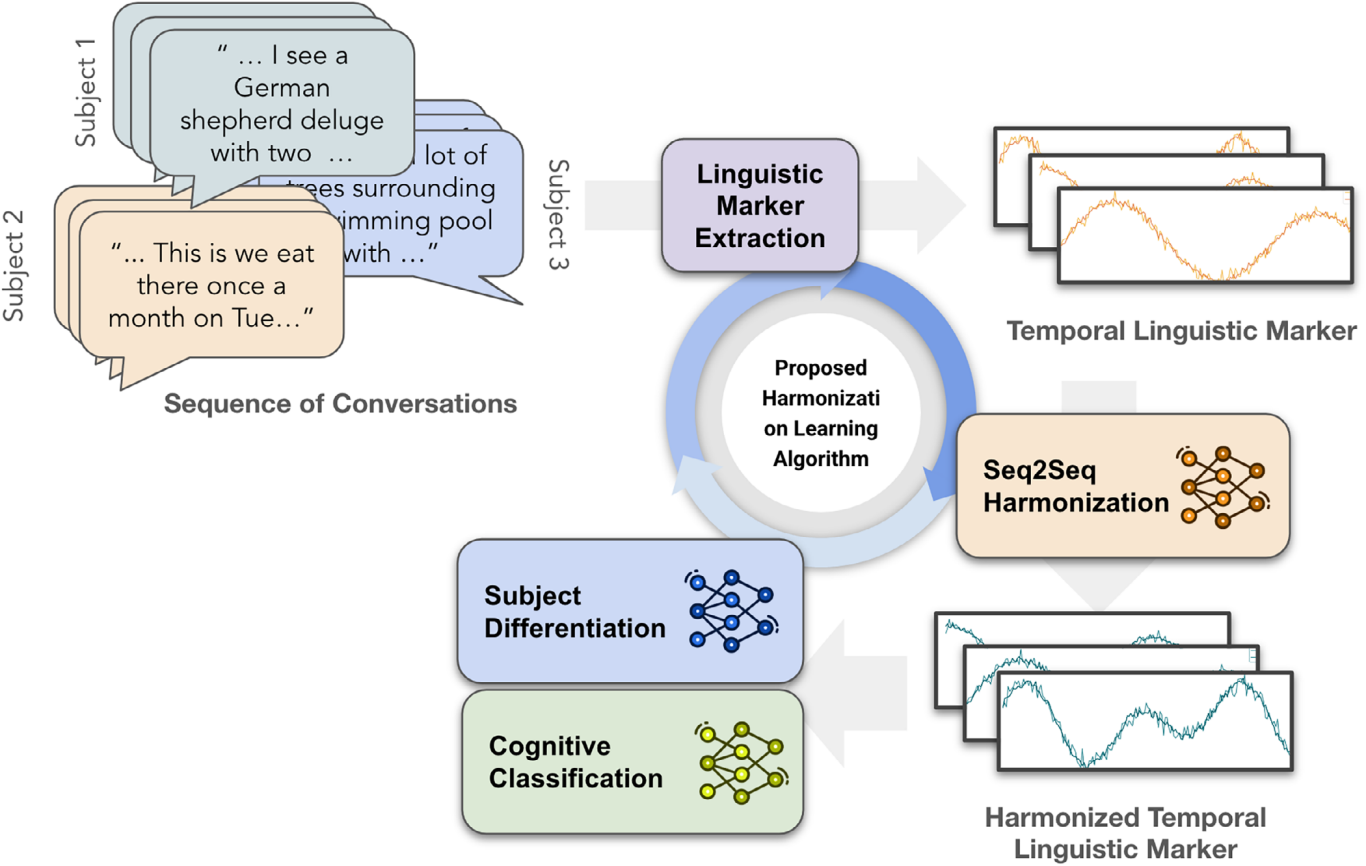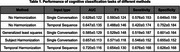# Improved Detection of Mild Cognitive Impairment from Temporal Language Markers Using Subject‐invariant Representation Learning using I‐CONECT data

**DOI:** 10.1002/alz70863_110588

**Published:** 2025-12-23

**Authors:** Bao Hoang, Yijiang Pang, Siqi Liang, Hiroko H Dodge, Jiayu Zhou

**Affiliations:** ^1^ Michigan State University, East Lansing, MI USA; ^2^ University of Michigan, Ann Arbor, MI USA; ^3^ Harvard Medical School, Boston, MA USA; ^4^ Massachusetts General Hospital, Harvard Medical School, Boston, MA USA

## Abstract

**Background:**

Mild Cognitive Impairment (MCI) is the prodromal stage of Alzheimer's disease. Early detection of MCI is essential for effective intervention and treatment. Recent studies have shown that linguistic marker is a promising and cost‐effective approach to identify MCI. Existing studies aggregated multiple rounds of conversation and demonstrated their effectiveness, and yet the use of finer granularity of conversation is not widely studied. Intuitively, the time series of language markers reveal important dynamics that are easily wiped off by aggregation. Significant individual differences in speaking styles pose challenges for sequence models in capturing cognitive characteristics. We propose a novel temporal harmonization method that mitigates distributional differences in temporal language markers across subjects, enhancing the prediction.

**Method:**

We utilized 6,771 conversations from 74 participants in Internet‐Based Conversational Engagement Clinical Trial (I‐CONECT) (NCT02871921). From each 30‐minute conversation session, we extracted a 99‐dimensional feature set including Linguistic Inquiry and Word Count, Syntactic Complexity, Lexical Diversity, and Response Length. For each subject, we have a sequence of feature vectors over time, up to 12 months. Our harmonization method leverages adversarial training via a min‐max optimization framework with three components: Seq2Seq Harmonization, a Subject Classifier, and a Cognitive Classifier. The harmonization module learns to remove subject‐specific components from temporal linguistic features, while the Subject Classifier infers subject identity. Finally, the Cognitive Classifier focuses on detecting MCI from the harmonized temporal features.

**Result:**

We compared our approach with single‐conversation input methods, where we trained neural networks to predict the outcome of individual conversations and used majority voting to determine the final output for each participant. Our results show that using temporal sequences improves detection performance compared to aggregated single‐conversation outputs, both with and without harmonization. When applying temporal harmonization, the performance of subject classification significantly increases, achieving an AUC of 0.720 compared to 0.647 without harmonization.

**Conclusion:**

Using only features extracted from semi‐structured conversation, we achieved a reasonalbe AUC. Our study demonstrates the additional benefits of using temporal sequences of language markers to detect Mild Cognitive Impairment. Moreover, applying temporal harmonization helps remove subject‐specific components in features and further enhances cognitive detection performance.